# Risk factors of non-sentinel lymph node metastasis in 443 breast cancer patients with sentinel lymph node-positive

**DOI:** 10.1097/MD.0000000000029286

**Published:** 2022-07-22

**Authors:** Shuang-long Cai, Ran-mei Wei, Lei Han, Xiao-geng Chen, Guo-xian Gong, Xiu-quan Lin, Jin Zhang, Hong-dan Chen

**Affiliations:** a Department of Oncological Surgery, Fujian Provincial Hospital, Fuzhou, China; b Department of Breast Disease, Qiqihar Traditional Chinese Medicine Hospital of Heilongjiang Province, Qiqihar, China; c Department of Ultrasonic Diagnosis Deparment, Fujian Provincial Hospital, Fuzhou, China; d Fujian Center for Disease Control and Prevention, Fuzhou, China; e Third Department of Breast Cancer, Tianjin Medical University Cancer Institute & Hospital, National Clinical Research Center for Cancer, Tianjin Key Laboratory of Cancer Prevention and Therapy, Tianjin's Clinical Research Center for Cancer, Key Laboratory of Breast Cancer Prevention and Therapy, Tianjin Medical University, Ministry of Education, Tianjin, China; f Fujian Provincial Hospital, Fuzhou, China.

**Keywords:** breast cancer, non-sentinel lymph node metastasis, risk factors, sentinel lymph node positive

## Abstract

Axillary lymph node dissection is the standard surgical procedure for breast cancer patients with sentinel lymph node (SLN) positive. In clinical practice, axillary lymph node dissection may be an unnecessary treatment for some breast cancer patients with non-sentinel lymph node (NSLN) negative. The aim of this study was to analyze the risk factors of NSLN metastasis in breast cancer patients with SLN positive. Four hundred fifty-six clinical early stage breast cancer patients with SLN positive were collected and analyzed in the oncological surgery department of Fujian Provincial Hospital during 2013 to 2018. All these patients underwent surgical treatment. The average age and tumor size of 443 patients with SLN positive breast cancer were (49.8 ± 10.8) years and (2.42 ± 0.94) cm. Univariate analysis showed that the size of primary tumor, the number of positive SLN, the number of negative SLN, the ratio of positive SLNs, and the type of metastases in SLN were the influencing factors of NSLN metastasis. Multivariate regression analysis showed that primary tumor size T > 2 cm (*P* < .001, OR = 2.609), the positive number of SLNs ≥3 (*P* = .002, OR = 5.435), the ratio of positive SLNs ≥ 50% (*P* = .017, OR = 1.770), and SLN macrometastases (*P* < 0.001, OR = 16.099) were independent risk factors for NSLN metastasis. Combined with the 4 independent risk factors, the area under the curve to predict NSLN metastasis was 0.747 > 0.7. For clinical early breast cancer with positive SLN, primary tumor size T > 2 cm,the positive number of SLNs ≥ 3, the ratio of positive SLNs ≥ 50%, and SLN macrometastases could predict NSLN metastasis well, and guide surgery to avoid overtreatment.

## 1. Introduction

The Global Cancer Statistics 2020 shows that female breast cancer has surpassed lung cancer as the number 1 in morbidity and number 2 cancer in mortality, with an estimated 2.3 million new cases (11.7%), followed by lung (11.4%).^[1]^ Based on the high incidence and fatality rate, breast cancer seriously harms the physical and mental health of women. Therefore, the precise treatment of breast cancer is an inevitable development trend.

Axillary lymph node status is an important component of breast cancer surgery and also considered as one of the most important prognostic factors.^[2]^ More than 2 decades ago, the biopsy of the sentinel lymph node (SLN) has safely replaced axillary lymph node dissection (ALND) for axillary staging in operable primary breast cancer surgery.^[3,4]^ If the SLN is histologically free of tumor cell, ALND could be omitted. However ALND still commonly performed after a positive SLN biopsy.^[5,6]^ However many scholars challenge this paradigm and believe that ALND for patients with positive SLNs may be an excessive treatment, since 40% to 70% of breast cancer patients with SLN metastasis do not have non-sentinel lymph node (NSLN) metastasis.^[7–9]^ The purpose of our study was to analyze the risk factors associated with NSLN metastasis in SLN-positive breast cancer patients.

## 2. Materials and Methods

### 2.1. Patients

The medical records of early breast cancer patients treated at the Fujian Provincial Hospital from January 2013 to January 2020 were retrospectively reviewed. This study was approved by the Ethics Committee of Fujian Provincial Hospital. The inclusion criteria were early breast cancer diagnosed by preoperative core needle biopsy or intraoperative frozen section analysis;) clinical physical examination and imaging examination showed clinical axillary lymph node-negative; no prior use of neoadjuvant chemotherapy or endocrine therapy; successful SLN biopsy. SLN positive patients undergo ALND. SLN-positive patients who did not undergo ALND were excluded.

**Figure 1.**
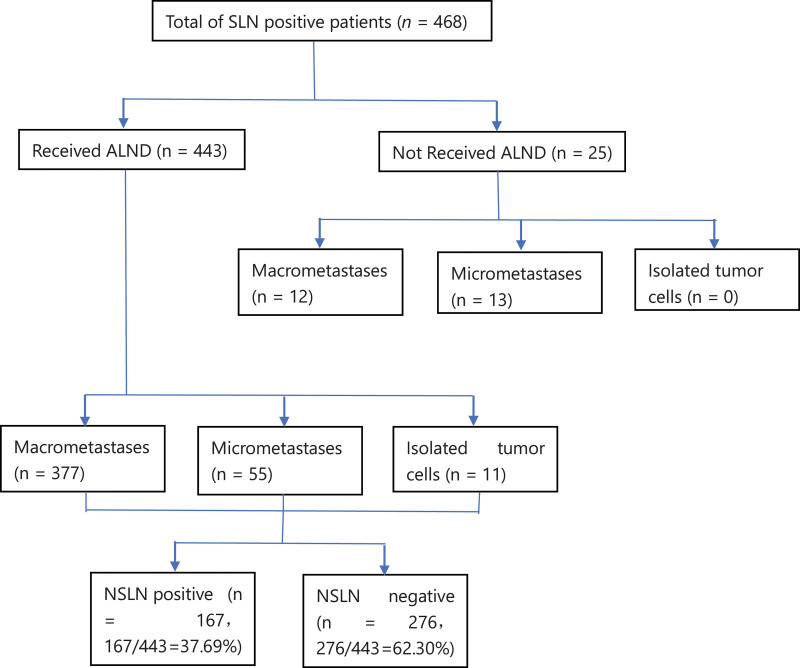
Schematic representation of nodal status.

Sentinel lymph node biopsy (SLNB) was successfully performed in 2488 patients, and positive SLNs were identified in 468 patients (Fig. 1). A total of 443 patients who met the inclusion and exclusion criteria were included in our study. Among the 443 patients, 377 patients had SLN macrometastases, 55 patients had SLN micrometastases, and 11 patients had found isolated tumor cell (ITC) in SLNs. Twenty-five SLN-positive patients did not undergo ALND. Among these 25 patients, 12 patients had SLN macrometastases, and 13 patients had SLN micrometastases.

Clinicopathological characteristics of patients in the study group were recorded, including age, tumor size, molecular classification, pathological type, histological grade, percentage of lymphocyte infiltration in tumor interstitial, lymphovascular invasion, the positive number of SLN, the negative number of SLN-negative, the positive rate of SLN metastasis, and the type of SLN metastasis. Molecular classification were classified into luminal A, luminal B, triple-negative and human epidermal growth factor receptor-2-positive. These were accorded to estrogen receptor, progesterone receptor, human epidermal growth factor 2 status, and the KI-67 index. All patients in the study received systematic adjuvant therapy in accordance with the National Comprehensive Cancer Network guidelines. Informed consent was obtained from all patients.

### 2.2. Sentinel lymph node biopsy

In patients for whom breast-conserving surgery or total mastectomy were planned, SLN biopsy were carried out with 0.5 mL of 1% methylene blue and nano-carbon into the subcutaneous tissue periareolar subcutaneous injection during the intraoperative period. Following the injection, all the patients received a breast massage for 5 to 10 minutes. Then, SLN biopsy was performed with a 2-cm radial incision between the outer edge of pectoralis major muscle and the anterior edge of latissimus dorsi muscle. At the end of stained lymphatic vessels, all of the lymph nodes that were blue and black-stained were accepted as SLN. The removed SLNs were examined by intraoperative rapid frozen section analysis and postoperative hematoxylin and eosin staining. According to the American Joint Committee on Cancer Staging Systems (8th Edition), SLN metastases were defined as macrometastasis (pN1, metastasis size >2 mm), micrometastasis (pN1mi, metastasis size between >0.2 mm and ≤2 mm), or ITCs (pN0[i+], metastasis size ≤0.2 mm).

### 2.3. Statistical analysis

Statistical analyses were conducted using SPSS version 22.0. Univariate analysis used *t* test, X^2^ test and Fisher exact test. *t* test was used for comparison of quantitative indicators between 2 groups, and X^2^ test and Fisher exact test were used for comparison of sample rates between 2 groups. Multivariate analysis used stepwise logistic regression, and all variables with a *P* value < .05 were included in the univariate analysis. In order to assess the predictive value of the multivariate logistic regression model, we used ROC to evaluate the independent risk factors obtained by the multivariate logistic regression.

### 2.4. Ethics Statement

This study was approved by the Ethics Committee of Fujian Provincial Hospital. Informed consent was obtained from all patients included in the study.

## 3. Results

**Figure 2.**
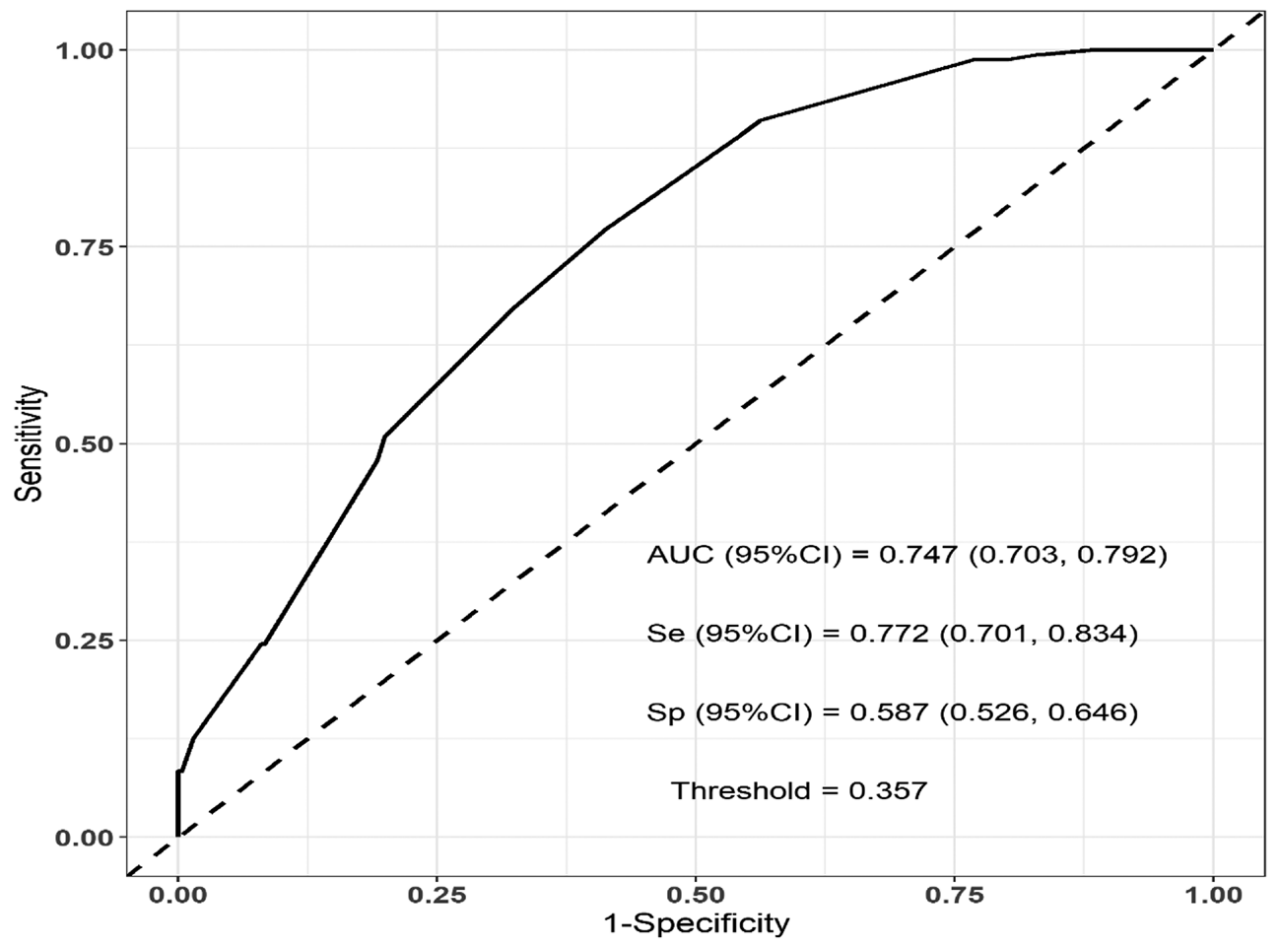
ROC curve of the combined with the 4 independent risk factors.

Two hundred seventy-six patients (62.30%, 276/443) were detected no metastasis in NSLN, while 167 patients (37.69%, 167/443) were detected metastasis in NSLN. In univariate analysis (Table 1), the size of primary tumor, the number of positive SLN, the number of negative SLN, the ratio of positive SLNs, and the type of metastases in SLN were associated with NSLN metastasis (*P* < .05). In multivariate analysis (Table 2), primary tumor size T >2 cm,the positive number of SLNs ≥3, the ratio of positive SLNs ≥50%, and SLN macrometastases were independent risk factors for NSLN metastasis. Combined with the 4 independent risk factors, the area under the curve to predict NSLN metastasis was 0.747 > 0.7 (Fig. 2).

**Table 1:** Analysis of risk factors for metastasis to non-SLNs in 443 SLN-positive patients.

	Non-SLNs				
Variables	Negative, n (%)	Positive, n (%)	Total	t/X^2^	*P* value
Age (yrs)	49.70 ± 10.72	49.78 ± 10.05	49.73 ± 10.46	#150;0.081	.936
Tumor size				24.690	
T =2 cm	168 (60.87)	61 (36.53)	229 (51.69)		
T >2 cm	108 (39.13)	106 (63.47)	214 (48.31)		
T staging					
T1	168 (60.87)	61 (36.53)	229 (51.69)		
T2	107 (38.77)	104 (62.28)	211 (47.63)		
T3	1 (0.36)	2 (1.20)	3 (0.68)		
Type of surgery				0.412	.521
Mastectomy	230 (83.33)	143 (85.63)	373 (84.20)		
Breast conserving	46 (16.67)	24 (14.37)	70 (15.8)		
Pathological type				0.001	.979
IDC	258 (93.48)	156 (93.41)	414 (93.45)		
Other	18 (6.52)	11 (6.59)	29 (6.55)		
Histological grade				3.945	.139
I	12 (4.35)	2 (1.20)	14 (3.16)		
II	241 (87.32)	147 (88.02)	388 (87.58)		
III	23 (8.33)	18 (10.78)	41 (9.26)		
Tumor stroma infiltrating lymphocyte				1.346	.718
0%#150;10%	209 (75.72)	129 (77.25)	338 (76.30)		
10%#150;20%	46 (16.67)	22 (13.17)	68 (15.35)		
20%#150;30%	12 (4.35)	9 (5.39)	21 (4.74)		
>30%	9 (3.26)	7 (4.19)	16 (3.61)		
Lymph-vascular invasion				0.157	.692
No	240 (86.96)	143 (85.63)	383 (86.46)		
Yes	36 (13.04)	24 (14.37)	60 (13.54)		
Number of sentinel lymph nodes detected				1.733	.188
1#150;2	100 (36.23)	71 (42.51)	171 (38.60)		
=3	176 (63.77)	96 (57.49)	272 (61.40)		
Number of sentinel lymph nodes negative					
	1.92 ± 1.62	1.31 ± 1.32	1.69 ± 1.54	4.078	
Number of sentinel lymph nodes positive					
	1.23 ± 0.46	1.63 ± 1.01	1.38 ± 0.75	#150;4.761	
Number of metastatic sentinel lymph nodes				27.845	
1	217 (78.62)	101 (60.48)	318 (71.78)		
2	54 (19.57)	45 (26.95)	99 (22.35)		
=3	5 (1.81)	21 (12.57)	26 (5.87)		
The ratio of positive sentinel lymph nodes				17.596	
	137 (49.64)	49 (29.34)	186 (41.99)		
=50%	139 (50.36)	118 (70.66)	257 (58.01)		
Sentinel lymph nodes transfer size				39.737	
ITC	11 (3.99)	0 (0.00)	11 (2.48)		
Micro	53 (19.20)	2 (1.20)	55 (12.42)		
Macro	212 (76.81)	165 (98.80)	377 (85.10)		
Estrogen receptor status	2.469	0.116			
Negative	36 (13.04)	31 (18.56)	67 (15.12)		
Positive	240 (86.96)	136 (81.44)	376 (84.88)		
Progesterone receptor status				3.254	.071
Negative	41 (14.86)	36 (21.56)	77 (17.38)		
Positive	235 (85.14)	131 (78.44)	366 (82.62)		
Human epidermal growth factor 2 status				0.008	.928
Negative	247 (89.49)	149 (89.22)	396 (89.39)		
Positive	29 (10.51)	18 (10.78)	47 (10.61)		
Ki-67 index	0.005	0.944			
=14%	39 (14.13)	24 (14.37)	63 (14.22)		
>14%	237 (85.87)	143 (85.63)	380 (85.78)		
Molecular subtypes				2.858	.414
Luminal A	38 (13.77)	24 (14.37)	62 (14.00)		
Luminal B	200 (72.46)	112 (67.07)	312 (70.43)		
Her2	15 (5.43)	9 (5.39)	24 (5.42)		
TNBC	23 (8.33)	22 (13.17)	45 (10.16)		

Use *t* test for 2 independent samples: age group, tumor size group, number of sentinel lymph nodes negative group, and number of sentinel lymph nodes positive group; besides these 4 groups, the other groups were tested by Pearson chi-square.

ITC = isolated tumor cell, Micro = micrometastases, Macro = macrometastases, Her2 = Human epidermal growth factor 2, SLN = sentinel lymph node, TNBC = triple negative breast cancer.

**Table 2:** Multivariate analysis for clinicopathological risk factors of non-sentinel lymph node metastasis.

Variable	Estimate	Se	z	Wald	P	OR (95%CI)
Tumor size						
T1	Ref					
=T2	0.959	0.219	4.375	19.142		2.609 (1.698, 4.010)
Sentinel lymph nodes transfer size						
	2.779	0.724	3.840	14.745		16.099 (3.898, 66.489)
Number of metastatic sentinel lymph nodes						
1	Ref					
2	0.202	0.262	0.772	0.596	.440	1.224 (0.732, 2.046)
=3	1.693	0.534	3.172	10.063	.002	5.435 (1.910, 15.466)
The ratio of positive sentinel lymph nodes						
	ref					
=50%	0.571	0.238	2.397	5.745	.017	1.770 (1.110, 2.822)

## 4. Discussion

Surgery is an important treatment for breast cancer patients. ALND could provide accurately pathological staging and reasonable follow-up treatment plan. Therefore, ALND improves loco-regional control, and reduce the risk of recurrence and metastasis. At the end it ultimately improves the prognosis of breast cancer patients. However ALND is also associated with the high incidence of postoperative complications hampered quality of patients’ life, such as lymphedema, limitation of shoulder motion, persistent seroma, and iatrogenic nerve injuries.^[10–12]^ The emergence of SLNB technology can predict axillary lymph node status, and ALND is an unnecessary treatment for patients without axillary lymph node metastasis. The American College of Surgeons Oncology Group Z0011 trial further supported that the early breast cancer patients (cT1-2N0M0) with 1 or 2 involved SLNs may omit ALND if followed by postoperative adjuvant radiotherapy and systemic adjuvant therapy.^[13,14]^ However, the Z0011 trial had a strong selective bias when the patients were enrolled. The trial enrolled a large proportion of patients with good prognosis. 27.3% NSLN metastasis patients were found in the ALND group. Most SLN-positive patients cannot meet the criteria of Z0011 trial in China, because of the high proportion of mastectomy in China.^[15]^ Among the 443 breast cancer patients with SLN-positive in this study, 167 (37.69%, 167/443) had NSLN metastasis, which was consistent with the results of previous studies.^[16–18]^ While 276 patients (62.30%, 276/443) with no metastasis in NSLN still underwent ALND. Therefore, the purpose of this study is to accurately identify NSLN metastasis in breast cancer patients with SLN positive. For these NSLN metastasis patients perform ALND, while NSLN no metastasis patients is exempted from ALND and reduce the proportion of overtreatment.

Primary tumor size is an important indicator to reflect the characteristics of breast cancer. Traditional perspectives considers that the larger primary tumor size led to the greater risk of axillary lymph node metastasis. The likelihood relationship between tumor size and NSLN metastasis has been reported in many studies. Some scholars’ research found a close positive correlation between tumor size >2 cm and NSLN metastasis.^[19,20]^ In our study, 49.53% (106/214) breast cancer patients with primary tumor size >2 cm had NSLN metastasis. Univariate and multivariate analyses showed that primary tumor size >2 cm was an independent risk factor for NSLN metastasis. The incidence of NSLN metastasis in ≥T2 patients group was 2.609 times as much as that in T1 patients group (*P* < .05).

In our study, while the number of SLN positive detected by intraoperative frozen biopsy were 1–2, or ≥3, the number of patients with NSLN metastasis was 35.01% (146/417) and 80.76% (21/26), respectively. Our result was consistent with the related research reports.^[21,22]^ Multivariate analysis showed that SLN positive number ≥3 was an independent predictor of NSLN metastasis. The ratio of positive SLNs was an independent risk factor in multiple NSLN prediction models. The incidence of NSLN metastasis was 26.48% (49/185) in SLN positive rate <0.5 group, while the incidence of NSLN metastasis was 45.91% (118/257) in SLN positive rate ≥0.5 group. Multivariate analysis showed that SLN positive rate ≥0.5 was more likely to associate with NSLN metastasis. And SLN positive rate ≥0.5 was an independent predictor of NSLN metastasis. The incidence of NSLN metastasis in SLN positive rate ≥50% group was 1.770 times as much as that in SLN positive rate <0.5 group.

Many studies have shown that SLN metastasis size is an independent predictor of NSLN status.^[23–25]^ When SLN metastasis size is macrometastases (>2 mm), micrometastases (0.2 mm < micro ≤ 2 mm), and ITCs (≤0.2 mm), the NSLN positive rate is 48%, 23%, and 12.5%, respectively. In our study, when SLN metastasis size is macrometastases, micrometastases, and ITCs, the NSLN positive rate is 43.76% (165/377), 3.63% (2/55), and 0% (0/11), respectively. The reason maybe be related to the fact that 13 patients with SLN micrometastasis did not undergo ALND and they were not included in the group. At the end of our study, univariate and multivariate analysis showed that SLN metastasis size was an independent risk factor of NSLN metastasis.

In our study, no significant correlation was found between pathological type, histological grade, percentage of tumor stroma infiltrating lymphocyte, lymph-vascular invasion, estrogen receptor, progesterone receptor, human epidermal growth factor 2, Ki67, molecular subtypes, and NSLN metastasis, which is different from relevant literature reports.^[26–30]^ The purpose of this study is to provide an important reference value for surgeons considering whether SLN positive breast cancer patients can omit ALND or not. After univariate and multivariate analysis, we found that primary tumor size T >2 cm, positive number of SLN ≥3, positive rate of SLNs metastasis ≥50%, and SLNs macrometastases were the independent predictors of NSLN metastasis. Combined with the 4 independent risk factors, the area under the curve to predict NSLN metastasis was 0.747 > 0.7, which could predict the risk of NSLN metastasis well in SLN-positive breast cancer patients.

However, Our study also has several limitations: The patients enrolled in this study were a retrospective study from a single center, which may result in selective bias. The enrolled patients did not routinely perform breast MRI examination. Therefore it is not very clear whether multifocal or multicentric breast cancer lesions may increase NSLN metastasis or not. A combined technique of radionuclide combined with isothiocyanine or patent blue dye injection were recommend in SLNB according to National Comprehensive Cancer Network guidelines. Considering the radiation risk of radionuclides, we used the methylene blue and nano-carbon in SLNB. The number of triple negative breast cancer and human epidermal growth factor 2 positive breast cancer patients in this study is too small. We need to expand our sample sizes to analysis whether different molecular subtypes of breast cancer would affect NSLN metastasis after a positive SLN biopsy.

Some scholars have established some prediction models to predict NSLN metastasis in SLN positive breast cancer patients, such as Memorial Sloan-Kettering Cancer Center model (United States),^[31]^ and Stanford University model.^[32]^ In addition, some scholars established NSLN metastasis predictive mode using 1-step nucleic acid amplification technique to evaluate CK19 mRNA copy number in SLN. This prediction tool could help in decision for ALND.^[33]^ Considering that breast cancer patients in different countries or regions may affect the accuracy of prediction models, we expect that a prediction model for NSLN metastasis based on the data of Chinese breast cancer patients can be established in the future, especially that there is no accepted prediction model in China. We wish could accurately screen NSLN metastasis patients in China with this prediction model, and provide a reference for clinical treatment decisions.

## Acknowledgments

We thank all patients who participated in our study.

## Author contributions

Shuang-long Cai, Lei Han, and Xiao-geng Chen contributed conception and design of the study. Shuang-long Cai, Lei Han, Xiao-geng Chen, and Xiu-quan Lin developed the methodology. Xiao-geng Chen, Guo-xian Gong, Ran-mei Wei, and Xiu-quan Lin took part in the acquisition, analysis, and interpretation of the data. Shuang-long Cai, Lei Han, Xiao-geng Chen, and Jin Zhang wrote, reviewed, and/or revised the manuscript. Xiao-geng Chen and Hong-dan Chen supervised the study. All authors contributed to the article and approved the submitted version.

Conceptualization: Lei Han, shuang-long cai, xiaogeng chen, Xiao-geng Chen.

Data curation: Guo-xian Gong, shuang-long cai, Xiao-geng Chen, Xiu-quan Lin.

Formal analysis: Ran-mei Wei, shuang-long cai, Xiao-geng Chen, Xiu-quan Lin.

Funding acquisition: Xiao-geng Chen.

Investigation: Guo-xian Gong, shuang-long cai, Xiao-geng Chen, Xiu-quan Lin.

Methodology: Lei Han, xiaogeng chen, Xiu-quan Lin, Xiu-quan Lin.

Software: Guo-xian Gong, Ran-mei Wei, Xiu-quan Lin, Xiu-quan Lin.

Supervision: Jin Zhang, Jin Zhang, xiaogeng chen.

Validation: Hong-dan Chen, Jin Zhang.

Visualization: Hong-dan Chen, xiaogeng chen, Xiao-geng Chen.

Writing – original draft: Hong-dan Chen, Jin Zhang, shuang-long cai.

Writing – review & editing: Hong-dan Chen, Jin Zhang.
